# Comprehensive Analysis of the Carcinogenic Process, Tumor Microenvironment, and Drug Response in HPV-Positive Cancers

**DOI:** 10.3389/fonc.2022.842060

**Published:** 2022-03-22

**Authors:** Xiaorong Yu, Jiankai Xu, Dahua Xu, Xiaoman Bi, Hong Wang, Yanda Lu, Meng Cao, Wenxiang Wang, Zhizhou Xu, Dehua Zheng, Liyang Chen, Xiaodian Zhang, Shaojiang Zheng, Kongning Li

**Affiliations:** ^1^ College of Bioinformatics Science and Technology, Harbin Medical University, Harbin, China; ^2^ Key Laboratory of Tropical Translational Medicine of Ministry of Education, College of Biomedical Information and Engineering and Cancer Institute of the First Affiliated Hospital, Hainan Medical University, Haikou, China

**Keywords:** HPV, carcinogenic process, tumor microenvironment, prediction model, drug response

## Abstract

Human papillomavirus (HPV) is a common virus, and about 5% of all cancers worldwide is caused by persistent high-risk HPV infections. Here, we reported a comprehensive analysis of the molecular features for HPV-related cancer types using TCGA (The Cancer Genome Atlas) data with HPV status. We found that the HPV-positive cancer patients had a unique oncogenic process, tumor microenvironment, and drug response compared with HPV-negative patients. In addition, HPV improved overall survival for the four cancer types, namely, cervical squamous cell carcinoma (CESC), head and neck squamous cell carcinoma (HNSC), stomach adenocarcinoma (STAD), and uterine corpus endometrial carcinoma (UCEC). The stronger activity of cell-cycle pathways and lower driver gene mutation rates were observed in HPV-positive patients, which implied the different carcinogenic processes between HPV-positive and HPV-negative groups. The increased activities of immune cells and differences in metabolic pathways helped explain the heterogeneity of prognosis between the two groups. Furthermore, we constructed HPV prediction models for different cancers by the virus infection score (VIS) which was linearly correlated with HPV load and found that VIS was associated with drug response. Altogether, our study reveals that HPV-positive cancer patients have unique molecular characteristics which help the development of precision medicine in HPV-positive cancers.

## Introduction

Human papillomavirus (HPV) is an important carcinogen since the HPV proteins E6 and E7 are intimately related to the events that cause malignant transformation of HPV-infected cells (1, 2). A global case statistics reported that cancers caused by HPV infection account for at least 5% (3). Persistent high-risk HPV infection can cause cancer in many different anatomical sites including cervix, penis, head and neck, lungs, prostate, bladder, and breast (4–11). Therefore, HPV has received more and more attentions as an independent carcinogen.

Present pan-cancer studies mainly focus on the impact of HPV on the tumor immune microenvironment, and most of them explain the possible benefits of HPV infection to patients from the perspective of immunotherapy. Gameiro et al. explained that the antitumor immunity activated by HPV might be the main source that improved the prognosis of HPV-positive patients in head and neck squamous cell carcinoma and such patients were suitable for immunotherapy (12). Varn et al. highlighted the changes in tumors caused by diverse virus infections and suggested that different families of viruses should be distinguished when designing immunotherapy methods (13). Cao et al. stated that viruses might help tumors escape the *PD-1* immune checkpoint pathway in multiple cancer types (14). Tumorigenesis depends not only on the alterations of the tumor microenvironment but also on gene mutations and the synergy of multiple carcinogenic pathways (15–17). However, those studies considered neither the differences of the carcinogenic processes between the HPV infection and other elements nor the possible impact of the expression level of HPV. Therefore, there is an urgent need for comprehensive and detailed analyses on the carcinogenic process, tumor microenvironment, and even the treatment outcome affected by both HPV and its expression level.

Here, we analyzed a total of 3,542 human samples representing 10 different cancers to describe how HPV caused cancers and shaped the tumor microenvironment at the genomics and transcriptomics level in The Cancer Genome Atlas (TCGA). Survival analysis showed that HPV played an important role for patients’ prognosis. Furthermore, we analyzed the differences in the carcinogenic processes between HPV-positive and HPV-negative groups from three aspects: driver genes, genome instability, and mitotic carcinogenic pathways. The results implied that HPV might trigger cancer through the cell-cycle disorder rather than genome instability. The tumor microenvironment is significantly related to the improvement of cancer patient survival and treatment effect (18, 19). In order to explain why HPV-infected patients’ survival was better than that of non-infected patients, we explored the impact of HPV on immune cell infiltration and metabolic pathway activity in the tumor microenvironment. We also found that the differences in the carcinogenic process and the tumor microenvironment mostly tended to appear in the tumor types with high HPV expression level. Next, we constructed HPV status prediction models to yield a virus infection score (VIS) for each cancer. VIS was positively correlated with HPV expression, and the classification efficiency of VIS was verified by both internal data from TCGA and external data from Gene Expression Omnibus (GEO). These models were also extended to Genomics of Drug Sensitivity in Cancer (GDSC) data and yielded VIS which represented the HPV-like status of GDSC cell lines. The higher VIS was related to the chemotherapy effect of TCGA patients and the drug sensitivity in the GDSC cell lines. In general, our research will help researchers to better understand the impact of HPV on the host genome and tumor microenvironment, and it will also be helpful in chemotherapy and immunotherapy for tumor patients with high HPV expression.

## Materials and Methods

### Datasets

TCGA samples were collected from the UCSC Xena pan-cancer project (http://xena.ucsc.edu/). The expression data were transcript per million (TPM) values with log_2_(x+0.001) transformed, and non-silent mutation was defined as gene-level mutation calls, where 1 represents non-silent mutation and 0 represents wild type. HPV expression (normalized reads per million, NRPM) was collected from a previous study (20), and the samples with more than 10 NRPMs were defined as infected by HPV. Only tumor types with at least 10 HPV-positive samples were considered, including cervical squamous cell carcinoma (CESC), uterine corpus endometrial carcinoma (UCEC), colon adenocarcinoma (COAD), rectum adenocarcinoma (READ), glioblastoma multiforme (GBM), ovarian serous cystadenocarcinoma (OV), esophageal carcinoma (ESCA), stomach adenocarcinoma (STAD), head and neck squamous cell carcinoma (HNSC), and kidney renal clear cell carcinoma (KIRC). At last, we collected 3,254 tumor samples in total (**Supplementary Tables S1, S2** ) with matched clinical data and chemotherapy response data from previous studies (21, 22). The driver gene and viral integration site for HNSC were collected from two other studies (23, 24).

External data with HPV status for model validation were obtained from the gene expression omnibus (GEO) with accession numbers GSE117973 and GSE151666. Cell line expression data and drug sensitivity data were downloaded from Genomics of Drug Sensitivity in Cancer (GDSC: https://www.cancerrxgene.org/, release8.2).

### Survival Analyses

The log-rank test was performed to evaluate the prognosis difference between HPV-positive and HPV-negative patients in each cancer type. In order to further explore the importance and impact of HPV on patient survival compared with other common clinical indicators, we used multivariate Cox regression for HPV status, age, gender, clinical stage, and TNM staging. Next, we performed stepwise regression based on the Akaike information criterion (AIC) to select variables which have important impact on patients’ survival. Survival analysis was performed by “survival” package in R.

### Calculation of Pathway Activity Scores and Collection of Immune Indicators

We collected gene sets for DNA damage repair (DDR) pathways (25), mitotic oncogenic pathways (17), and metabolic pathways (26). Pathway activity scores were calculated using the single sample gene set enrichment analysis (ssGSEA) method in R package “GSVA”. The abundance of immune cells were derived from xCell which was a gene signature-based method to quantify 64 cell types through ssGSEA (27).

Other genomic instability and immune indicators covering “loss of heterozygosity (LOH) frac altered,” “Aneuploidy Score”, “Copy Number Variation (CNV) burden”, “Microsatellite Instability (MSI) score”, “Mutation load”, “Cytolytic Activity (CYT) score”, “Cancer-testis Antigen (CTA) score”, “Neoantigens”, “B cell receptor repertoire (BCR)”, and “T cell receptor repertoire (TCR)” were obtained from previous studies (20, 28).

### Construction of the HPV Status Prediction Model

In order to observe the spatial proximity of these samples, we put all samples to a two-dimensional coordinate system using Uniform Manifold Approximation and Projection (UMAP) dimensionality reduction through the “umap” package and then clustering analysis was performed by the “dbscan” package.

The differential gene expression analysis between HPV-positive and HPV**-**negative samples within a given cancer type was performed by the “DEseq2” package (29). The genes with both |Log2 fold change| > 1 and adjusted p-value < 0.05 represented the different transcriptome features between the two groups. To increase reliability and accuracy for predicting HPV status, the lasso regression model was constructed for each cancer type with the sample’s HPV status as the response variable and the gene expression level as the predictor variable by the “glmnet” package. HPV signature gene sets (the predictor variables) were derived by stepwise regression and were used to calculate the virus infection score (VIS) by the corresponding lasso model in each cancer type. VIS was defined as the sum of (regression coefficient* signature gene expression level) in each sample (**Supplementary Table S3**). The relationship between VIS and NRPM was estimated by the Spearman correlation coefficient. AUC was calculated by the “pROC” package to verify the performance and ability of each cancer prediction model, and two external GEO datasets (GSE117973 and GSE151666) were further used. In addition, the prediction models were extended to GDSC cell line data to capture HPV-like infected samples which had similar transcriptomic features with HPV-positive cancer patients.

### Connection Between VIS and Drug Response

To evaluate the connection between VIS and drug response, we combined all cancer types’ VIS after z-score transformation and divided TCGA samples into four groups according to chemotherapy response: “complete response” (CR), “partial response” (PR), “stable disease” (SD), and “clinical progressive disease” (CPD). We explored the distribution of VIS in the four groups and calculated the proportion of chemotherapy response in different groups segmented by scaled VIS. GDSC data were divided into two categories according to the threshold of scaled VIS = 1, and the difference of the half maximal inhibitory concentration (IC50) was analyzed between the two categories.

### Statistical Analyses

Fisher’s exact test was used to evaluate the difference of gene mutation frequency between HPV-positive and HPV-negative groups. All the comparisons of pathway activity and other indicators between the two groups were performed by the two-tailed Wilcoxon-sum rank test. In the GDSC data set, the difference of IC50 between two groups was calculated by the two-tailed T test or Mann–Whitney U test when the data were not normally distributed. All statistical analyses were performed by R.

## Results

### HPV Improves Overall Survival for Four Cancer Types

**Figure 1:**
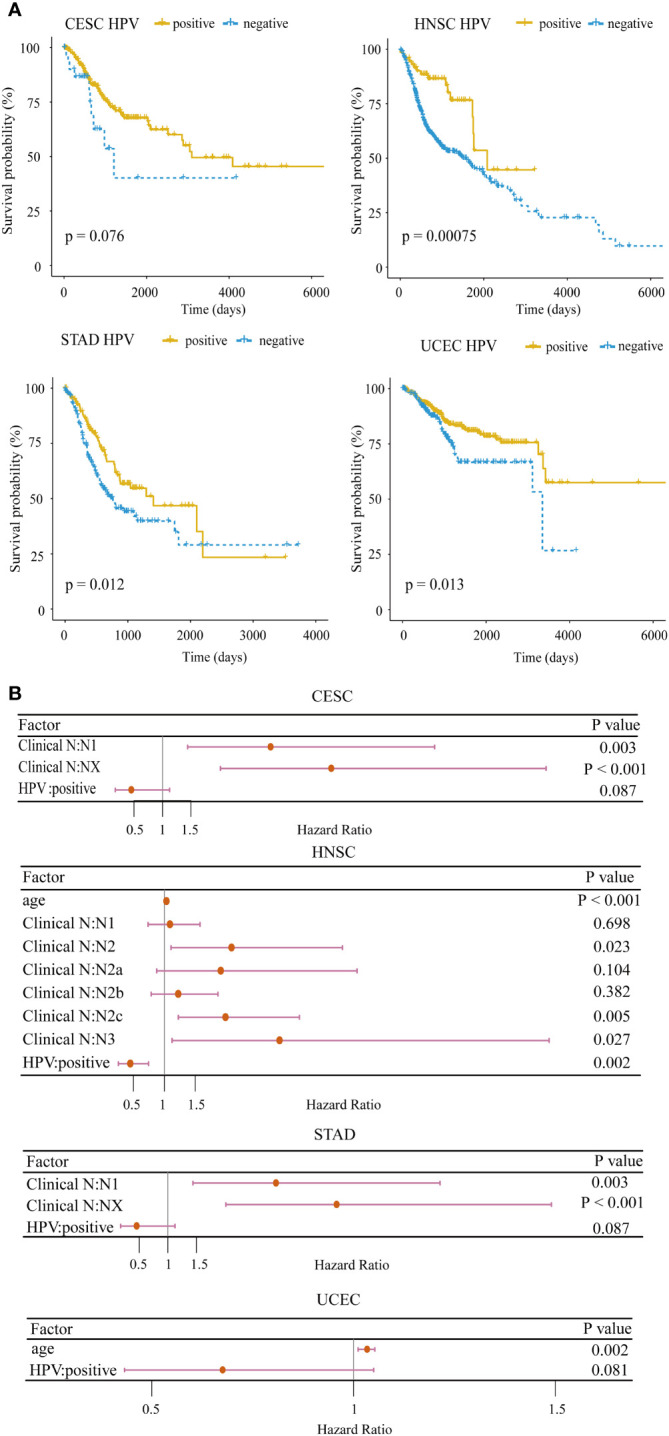
Survival analysis for HPV-related cancer. **(A)** The HPV-positive group had significantly higher survival rates compared with the HPV-negative group in four cancer types (CESC, cervical squamous cell carcinoma; HNSC, head and neck squamous cell carcinoma; STAD, stomach adenocarcinoma; UCEC, uterine corpus endometrial carcinoma). **(B)** Forest plot of the multivariate Cox regression analysis of HPV and clinical indicators with stepwise selection. The red horizontal lines correspond to the 95% CI, on which the dot reflects the hazard ratio. (Nx, regional lymph nodes could not be evaluated; N1, lymph node metastases with a maximum diameter of less than 3 cm; N2, lymph node metastases with a maximum diameter of less than 6 cm and greater than 3 cm; N3, the maximum diameter of metastatic lymph nodes is greater than 6 cm).

Clinically, HPV-positive patients in HNSC have a better overall survival than HPV-negative patients (30). To confirm the impact of HPV on the prognosis for HPV-related cancers, we applied the log-rank test to analyze the differences in the survival times between HPV-positive and HPV-negative groups. In 4/10 cancer types including CESC (p = 0.076), HNSC (p = 0.00075), STAD (p = 0.012), and UCEC (p = 0.013), HPV-positive patients exhibited a significantly better prognosis (**Figure 1A**). In particular, the survival rate of HPV-positive patients did not drop rapidly as HPV-negative patients within the first 5 years in HNSC. To further demonstrate the importance of HPV infection on patient survival, we examined the hazard ratio of HPV infection compared with other common clinical indicators through the multivariate Cox proportional hazard model among the above four cancer types. HPV remained as a favorable prognostic for the four cancer types after stepwise regression screening based on Akaike information criteria (**Figure 1B**). This result implied that HPV could be an indicator of patient prognosis, which was as important as the clinical stage. These analyses hint that HPV infection induces an underlying mechanism that makes the prognosis of hosts better than that of non-infected samples.

### HPV-Positive Patients Have Stronger Cell Cycle Activity in the Carcinogenic Process

**Figure 2:**
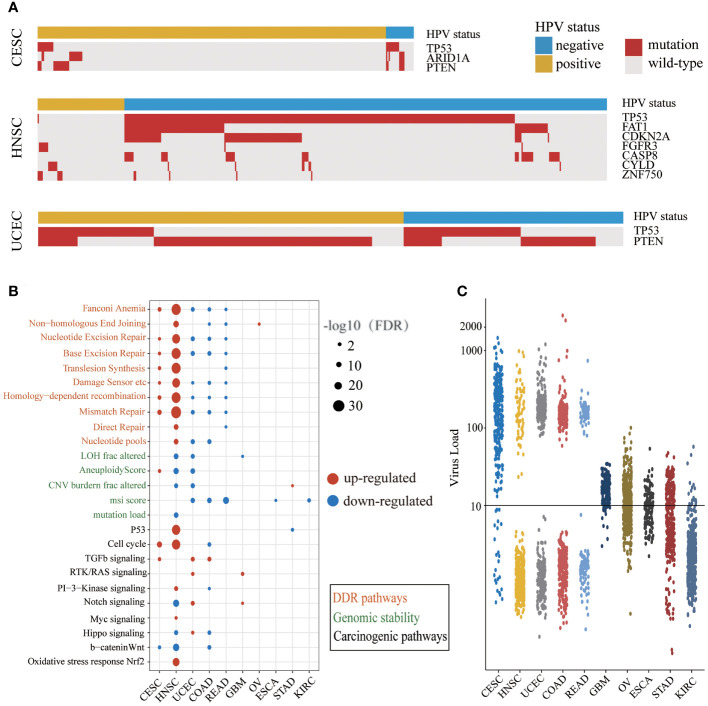
The different carcinogenic processes between HPV-positive group and HPV-negative group. **(A)** A waterfall plot showing the significant differences in driver gene mutations between the HPV-positive group and HPV-negative group in three cancer types (CESC, cervical squamous cell carcinoma; HNSC, head and neck squamous cell carcinoma; UCEC, uterine corpus endometrial carcinoma). The top panel shows the HPV status of each sample. Red boxes represent gene mutation, and while gray boxes represent wild-type. **(B)** A bubble plot shows the significant differences of DDR (DNA damage repair) pathway activity, genomic instability indicators, and mitotic oncogenic pathway activity between the HPV-positive group and HPV-negative group. The size of the bubble represents FDR, and the color represents upregulation or downregulation. The color of the label on the Y-axis represents the different carcinogenic processes. **(C)** The NRPM (normalized reads per million) of HPV in different cancer types. The threshold of HPV infection was signed by a black horizontal line.

The mutation frequencies of several driver genes in the HPV-positive group with CESC and HNSC were significantly lower than those in the HPV-negative group (**Figure 2A** and **Supplementary Table S4**). The lower mutation frequency of *TP53* in the HPV-positive group of CESC and HNSC indicated that their genomes were more stable. In the HPV-positive group, the lower mutation frequency of *ARID1A* in CESC as well as *FAT1*, *CDKN2A*, and *FGFR3* in HNSC demonstrated that abnormal cell proliferation of HPV-positive patients did not arise from driver gene mutations. Although *CYLD* and *ZNF750* mutations were enriched in HPV-positive HNSC, the samples with these two gene mutations together accounted for only 20% of HPV-positive ones. These results indicated that those genes in the HPV-positive group were not the same as *TP53* which was the main cause of cancer in the HPV-negative group. Additionally, *TP53* mutations in UCEC were enriched in the HPV-negative group, and *PTEN* mutations were enriched in the HPV-positive group. The total number of mutations with *TP53* or *PTEN* exceeded 80% in both HPV-positive and HPV-negative patients. This result illustrated that there was no difference in the driver gene level between HPV-positive and HPV-negative groups in UCEC. We further applied the t-test to compare the expression levels of different mutation driver genes with the HPV-negative group in the three cancer types. As shown in **Supplementary Figure S1**, the expression of *CASP8*, *CDKN2A*, *FGFR3*, and *TP53* group was higher in HPV-positive than those in HPV-negative group in HNSC. The expression of *PTEN* and *TP53* was higher in the HPV-positive group than those in the HPV-negative group in UCEC, but there were no differences in CESC.

We examined the differences of DDR pathway activity and other genomic instability indicators between HPV-positive and HPV-negative groups (**Figure 2B**). The DDR pathway activity in CESC and HNSC was enhanced in the HPV-positive group, which might be related to the less mutation of *TP53*. The DDR pathway activity was decreased in the HPV-positive group of UCEC, COAD, and READ, and there were a few changes in the rest of the cancer types. It is worth noticing that the alternation of indicators for genome instability was consistent with the DDR repair pathway activity only in HNSC. One possible explanation is that there may be other DDR repair mechanisms in addition to the 10 DDR repair pathways.

Next, we compared the difference in mitotic oncogenic pathways between HPV-positive and HPV-negative groups (**Figure 2B**). HNSC was the most affected cancer by HPV infection, and the activity of the P53 pathway was also related to the low mutation rate of the *TP53* gene in the HPV-positive group. In CESC and HNSC, HPV activated the cell cycle through different pathways like the PI3K and MYC signaling pathway in HNSC and the TGF-β signaling pathway in CESC. In addition, the cell-cycle activity of the HPV-positive group in COAD was lower than that of the HPV-negative group, indicating that the impact of HPV infection in this cancer was different from that in CESC and HNSC. In GBM, STAD, and UCEC, the cell-cycle activity did not change significantly, indicating that although individual mitotic oncogenic pathways of these cancers could be affected by HPV, it was not reflected in the cell cycle. In addition, we observed a considerable number of overlaps between DE genes and these essential pathways (**Supplementary Table S5**).

Interestingly, we noticed that the changes in the DDR and mitotic oncogenic pathways were related to HPV expression. The cancer types with large-scale variations in the DDR and carcinogenic pathway activity exhibited a high HPV expression level. In most cases of CESC, HNSC, UCEC, COAD, and READ, the NRPM value exceeded 100 or even reached 1,000 (**Figure 2C**). This phenomenon revealed that the impact of HPV on the host carcinogenic process might depend on its expression level. In summary, the carcinogenesis of the HPV-positive group in CESC and HNSC was triggered by the active cell cycle after HPV infection rather than genome instability, which was a major difference between HPV-positive and HPV-negative patients in CESC and HNSC.

### HPV Affects the Tumor Microenvironment

**Figure 3:**
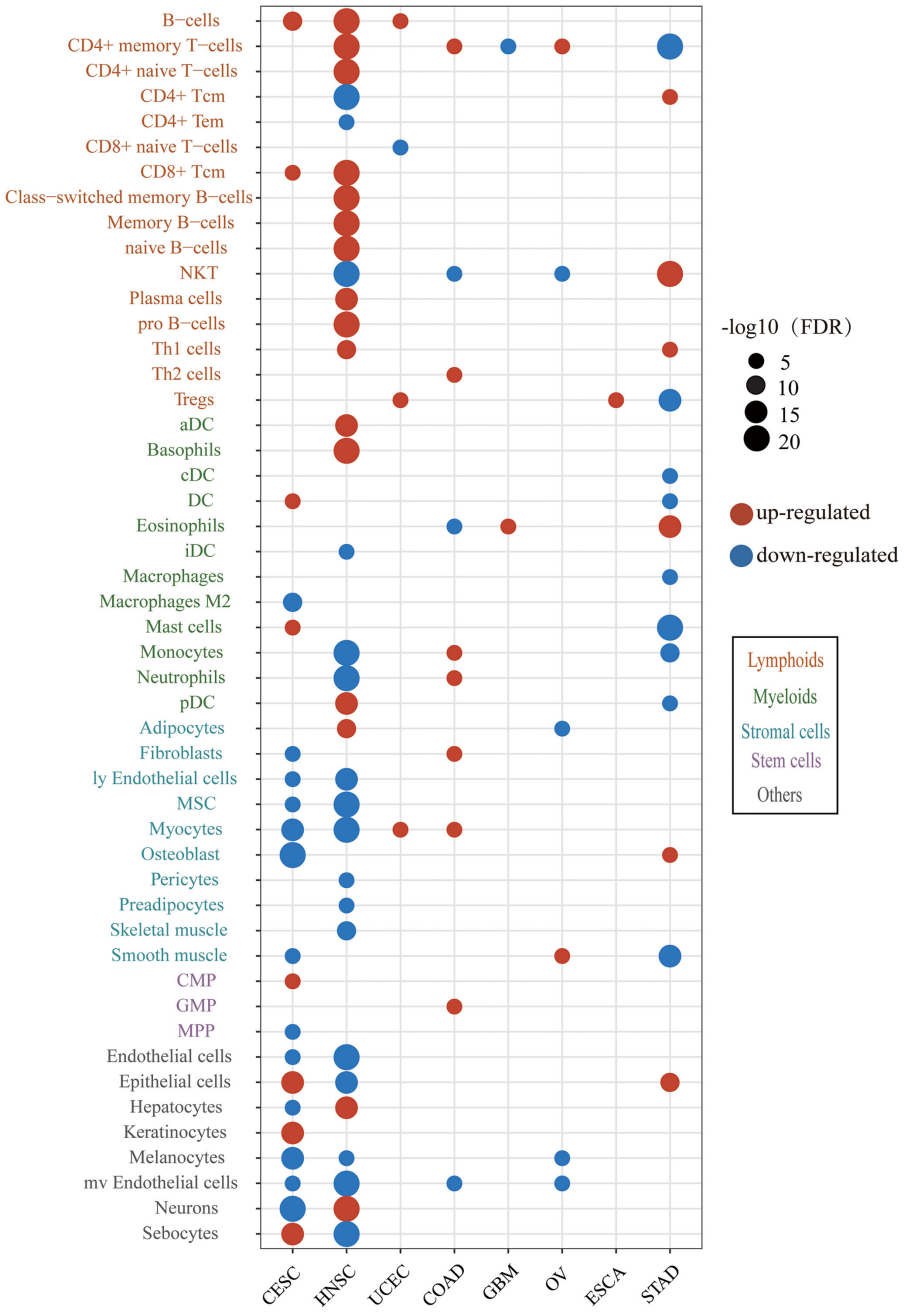
The difference of immune and stroma cell types between the HPV-positive group and HPV-negative group. The red bubbles represent the significantly upregulated abundance of immune cell infiltration, and the blue ones represent that significantly downregulated. The sizes of the bubbles represent FDR, and different cell types are marked by different colors on the Y-axis label (CESC, cervical squamous cell carcinoma; HNSC, head and neck squamous cell carcinoma; UCEC, uterine corpus endometrial carcinoma; COAD, colon adenocarcinoma; GBM, glioblastoma multiforme; OV, ovarian serous cystadenocarcinoma; ESCA, esophageal carcinoma; STAD, stomach adenocarcinoma).

To obtain insights into the immune infiltration affected by HPV, we examined the different abundance of immune cell infiltration between HPV-positive and HPV-negative groups. The results showed that HPV infection affected the tumor immune microenvironment in 8/10 cancer types and 49/64 cell types (**Figure 3**). The immune cell infiltration of HNSC was most widely affected by HPV. The HPV-positive groups of CESC and HNSC had the common characteristics of elevated B cells and CD8+Tcm infiltration. The upregulation of immune cell infiltration may be the reason for the better prognosis in HPV-positive patients, such as B cells and CD8+ Tcm in CESC, CD8+ Tcm in HNSC, NKT cells in STAD, and B cell in UCEC. These cells can directly or indirectly kill tumor cells. At the same time, the stromal cells of CESC and HNSC were decreased on a large scale, which was helpful to improve the prognosis of patients (31, 32).

**Figure 4:**
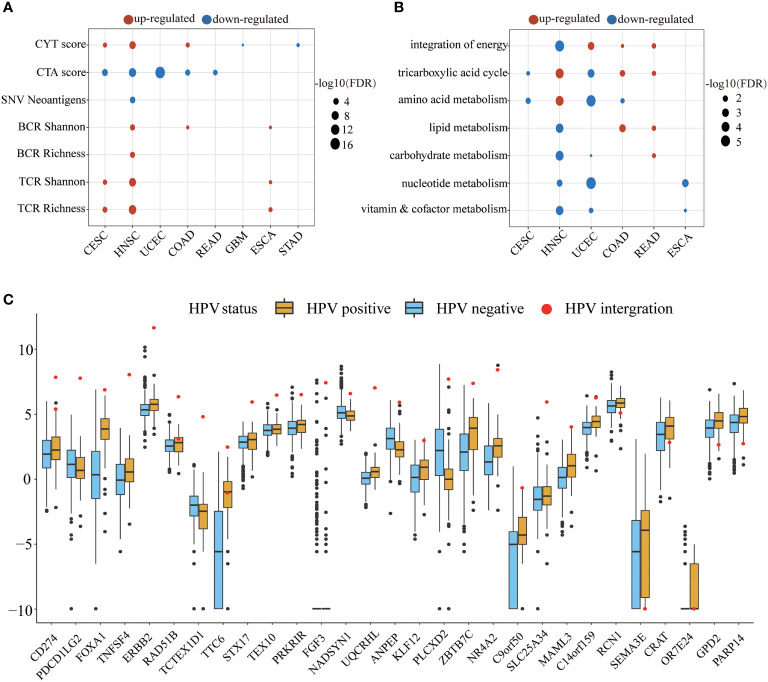
The HPV impact on the tumor microenvironment. **(A, B)** show the significant changes in immune indicators and metabolic pathway activity, respectively. The size of bubble represents FDR, and the color represents upregulation or downregulation. All the p values of the non-parametric test have been corrected by FDR. **(C)** The figure shows significantly different expression of HPV-integrated protein-coding genes between the HPV-positive group (yellow) and the HPV-negative group (blue). The red dots in the box diagram are the expression of HPV-integrated genes in HNSC.

We next examined the alteration of immune indicators by HPV infection (**Figure 4A**). The CYT score increased in the HPV-positive group of CESC, HNSC, and COAD, indicating that HPV stimulated the enhancement of the cytotoxic T cells (CTL) of these three types of cancer. Studies have shown that cancer-testis antigen (CTA) contributes to tumorigenic signal transduction (33), and it has been regarded as a potential target of treatment (34, 35). The CTA score in HPV-positive patients of CESC, HNSC, COAD, READ, and UCEC were decreased, implying that the treatment strategy for the CTA antigen might not work for these cancers. The reduction of neoantigens in the HPV-positive group of HNSC may be due to its lower mutation load. TCR is responsible for the detection of human “non-self” antigens (36). The increased TCR in the HPV-positive group of CESC, HNSC, and ESCA meant enhanced ability of T cell recognition. The higher BCR of the HPV-positive group in HNSC, COAD, and ESCA also indicated that HPV as a foreign substance stimulated the activation of the host humoral immune system.

The metabolic pathways were also affected by HPV infection. HNSC, UCEC, COAD, and READ patients received the energy for tumor cell growth through at least one metabolic pathway for the integration of energy or the tricarboxylic acid cycle. The upregulation of carbohydrate metabolism, nucleotide metabolism, and vitamin and cofactor metabolism metabolic subtypes is always associated with poor prognosis (37). The downregulation of these pathways in the HPV-positive group of HNSC and UCEC may be another reason for their better prognosis.

The integration of HPV DNA into the host genome is an important event that leads to abnormal proliferation and malignant progression during HPV-mediated carcinogenesis (38, 39). The NHEJ (non-homologous end joining) pathway was more active in the HPV-positive group of HNSC (FDR = 2.84E-09), which provided the necessary conditions for HPV integration. HPV-integrated coding genes in HNSC tended to be enriched in GO terms that negatively regulated the host’s immune response and cell adhesion (**Supplementary Figure S2**). Among the 60 HPV-integrated protein-coding genes, 47 genes were upregulated and 13 were downregulated according to the Tukey standard. The expression of HPV-integrated genes was increased abnormally in HPV-positive patients, which included the famous immune checkpoint genes *CD274* and *PDCD1LG2* (**Figure 4C**).

### Construction of the HPV Status Prediction Model by Transcriptome Characteristics

**Figure 5:**
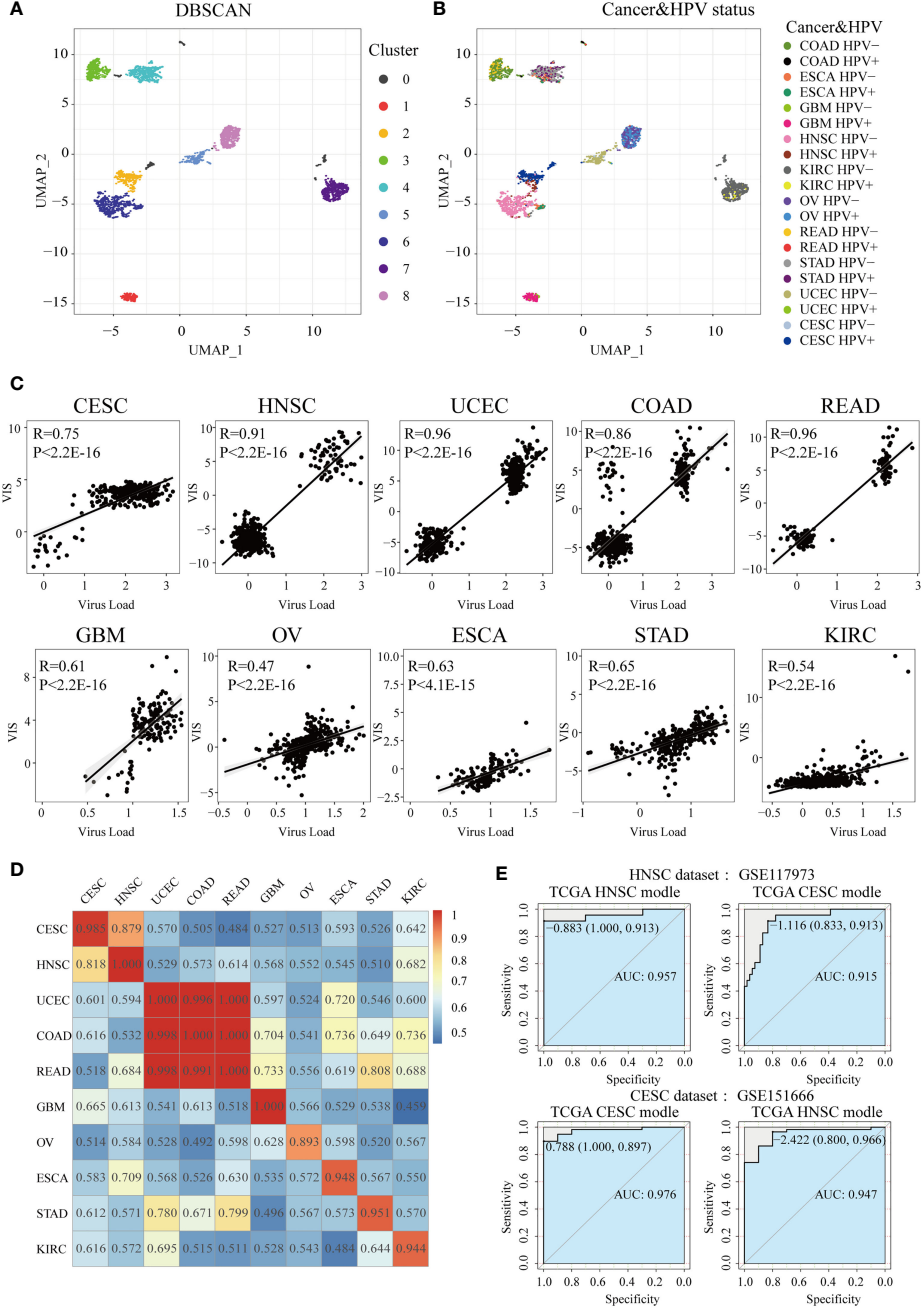
Construction of HPV the prediction model. After UMAP (Uniform Manifold Approximation and Projection) dimensionality reduction, all samples are projected to a two-dimensional coordinate system. The colors of the points in **(A)** and **(B)** represent the different clusters after DBSCAN (Density-Based Spatial Clustering of Applications with Noise) clustering and HPV status in different cancer types, respectively. **(C)** The Spearman correlation between VIS and NRPM in each cancer type. **(D)** A heatmap shows the AUC values of prediction models which were trained in one cancer type (rows) and applied to the others (columns). **(E)** Two GEO data sets were used for external verification of HPV status prediction models derived from TGCA HNSC and CESC data.

To explore the potential connection within and between tumor types of HPV-related cancer patients, we used the UMAP method to reduce the dimensionality of transcriptome in all samples and then projected samples into a two-dimensional coordinate system (**Figures 5A, B**). Cancer samples tended to cluster according to the cancer type and were also closer according to similar tissues among cancer types (the top-left corner were four types of pan-digestive tract cancers, and the center of the coordinate system was occupied by gynecological cancers). It is worth noting that CESC samples were close to HNSC samples and HPV-infected samples in HNSC tended to cluster with CESC, implying that HPV-positive samples in HNSC and CESC samples were relatively similar at the transcriptomic level (**Figure 5B**). It is reasonable that HNSC and CESC are both squamous cell carcinomas in terms of cell origin (40). This also explains the similar changes in several pathways and the tumor microenvironment in the HPV-positive group of the two cancers.

In order to construct and evaluate the performance of HPV prediction models based on HPV-related transcriptome features, we used lasso regression to screen differential genes related to HPV infection status, then obtained the signature gene set and prediction model of 10 cancers (**Supplementary Table S3**). Next, lasso regression combined with the signature gene sets was used to calculate the virus infection scores (VISs) for each sample. The VIS was significantly positively correlated with the NRPM value (correlation coefficients from 0.47 to 0.96, **Figure 5C**). We also applied the prediction model for a specific cancer type into other cancer types and used the AUC value to evaluate the accuracy of each model across cancer types (**Figure 5D**). The prediction model had the best efficiency in predicting the HPV infection status for its own cancer. However, some models still had high AUC values (AUC >0.90) when they were applied into other cancer types, such as the models established in CESC and HNSC as well as the models in COAD, READ, and UCEC (**Figure 5D**). High AUC values were still achieved when two sets of external data (HNSC: GSE117973, CESC: GSE151666) were used to verify the classification efficiency of the model (**Figure 5E**), resulting in that the models of CESC and HNSC were interchangeable (AUC >0.9).

### VIS Is Associated With Drug Response

**Figure 6:**
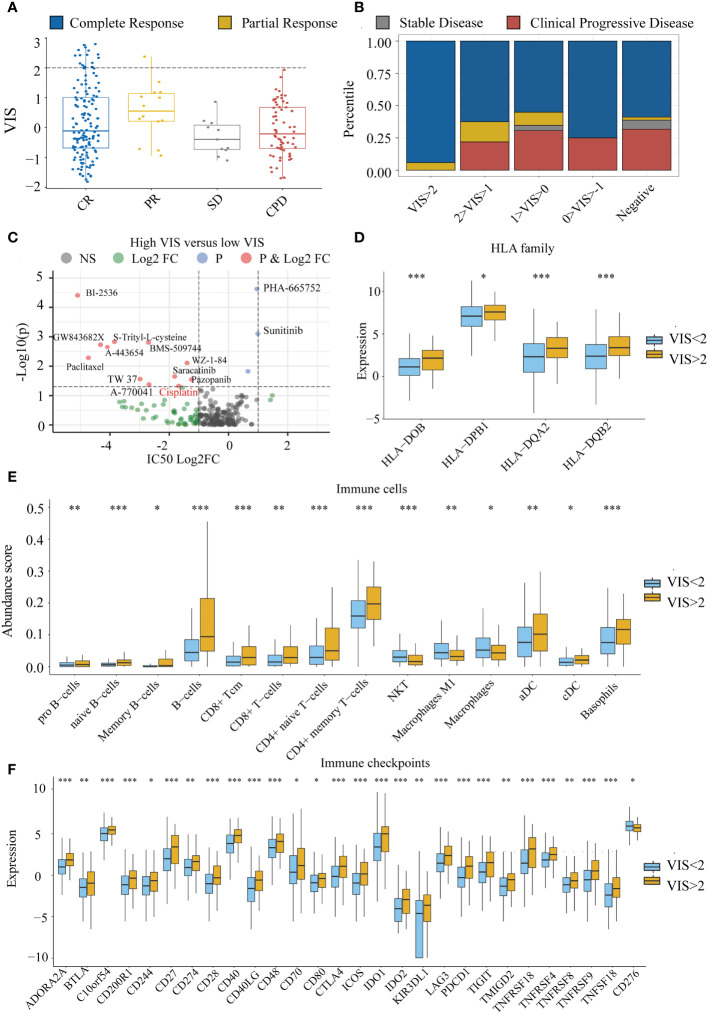
The connection between VIS and drug response. **(A)** Distribution of scaled VIS levels for TCGA stage III and stage IV samples according to the RECIST standard. **(B)** Proportion of chemotherapy response in different groups segmented by scaled VIS. **(C)** Differences in drug IC50 values between high- and low-scaled VIS groups in the GDSC cell line data. **(D–F)** Show the significant differences in HLA family expression, immune cell infiltration, and immune checkpoint gene expression between high- and low-scaled VIS groups. All the p values of nonparametric test have been corrected by FDR (* represent fdr < 0.05, ** represent fdr < 0.01 and *** represent fdr < 0.001).

To explore whether there is a relationship between VIS and drug response, we divided the TCGA samples into four groups according to the RECIST standard. We found that VIS was related to the chemotherapy response of the TCGA sample. When the scaled VIS >2, the rate of CR to the chemotherapy of the TCGA samples in stage III and stage IV was 94% without drug resistance (**Figures 6A, B**), and the ratio of CR was much greater than that of the scaled VIS <2 group (OR = 11.10, p = 0.0034). We also found that scaled VIS had a connection with drug response in the GDSC cell line data of high-confidence cancer types whose correlation coefficients between VIS and NRPM were greater than 0.8. Some drugs had lower IC50 values in the cell lines with high-scaled VIS, indicating that these drugs were more sensitive in high-scaled VIS cell lines (**Figure 6C**). Interestingly, more than half of samples (59%) in the CR group with scaled VIS >2 used platinum-based chemotherapy drugs in the TCGA dataset, and the lower IC50 value of cisplatin was also found in high-scaled VIS cell lines. We also found that the efflux gene *ATP7B* was significantly lower in the scaled VIS >2 group, which caused the different chemotherapy outcome by cisplatin (**Supplementary Figure S3**). There were no immunotherapy drugs in the above analysis, so we explored the feasibility of immunotherapy for scaled VIS >2 samples. Studies have shown that the HLA family (41), immune cells (42), and immune checkpoint (43) can affect immunotherapy. We found out the significantly increased expression of the HLA family, abundance of immune cells (except NKT, macrophages), and expression of immune checkpoints (except *CD276*) in the scaled VIS >2 group in TCGA data (**Figures 6D–F**). These differences indicate that patients with higher VIS may be likely to benefit from immunotherapy.

## Discussion

We have discovered that HPV contributes to favorable prognosis for CESC, HNSC, UCEC, and STAD (**Figure 1B**), implying that even if HPV is a carcinogen, it can also activate uncertain mechanisms of the host to prolong the survival. However, HPV viral load was not significantly correlated with overall survival (Spearman’s rank correlation, p = 0.2012). HPV-positive clinical associations were further analyzed by the chi-square test or the Fisher test in R (**Supplementary Table S6**). HPV-positive tumors were more associated with lower stages (staging in UCEC, pathological T in STAD and HNSC, pathological N in HNSC, and pathological M in CESC) than the HPV-negative tumors. The above associations were the composite effects of the carcinogenic process and the tumor microenvironment.

The HPV E6 and E7 oncoproteins are the dominant paradigm for tumorigenesis. The expression of E6 stimulates p53 degradation, while the expression of E7 degrades Rb, leading to an increase in E2F-dependent transcription and a deregulation of the cell cycle without control of DNA replication, DNA repair, and apoptosis (44). In HPV-positive cervical cancer cell lines, the knocked-down E6/E7 could increase p53 at the protein level, thus hindering cell growth and triggering cell death *in vitro* and *in vivo* (45). The significantly lower frequency mutation (**Figure 2A**) and higher expression (**Supplementary Figure S1**) of *TP53* in the HPV-positive group of HNSC and UCEC implied a more functional P53 expression and might explain in part increased chemosensitivity and radiosensitivity (46, 47). The loss control of the cell cycle induced by upregulated *E2F1* in the HPV-positive group of CESC, HNSC, and UCEC and downregulated *RB1* in the HPV-positive group of STAD played an important role on the formation and progression of the four cancers. The third oncoprotein E5 expressed together with two regulatory proteins (E1 and E2) attributes to p53-dependent enhanced proliferation *in vitro* and activates the *FGFR* pathway to accelerate tumorigenesis *in vivo* (48). Members of the *FGFR* family were upregulated in the HPV-positive group of the four cancers (**Supplementary Table S6**), implying combined inhibition of *FGFR* and *mTOR* for targeted therapy (48, 49).

We identified two cancer types, CESC and HNSC, whose driver genes were enriched in HPV-negative patients, especially the low-frequency mutation of *TP53* which was a common feature of HPV-positive patients in CESC and HNSC. The important role of the P53 protein can maintain genome stability (50, 51); thus, the activation of DNA damage repair pathways in HPV-positive patients of these two cancers was observed (**Figures 2A, B**). Mutated *ARID1A* can cause abnormal cell proliferation and block immune checkpoint therapy (52–54), and the co-occurrence of the lower mutation frequency of *ARID1A* and higher infiltration of CD8+Tcm may enable HPV-positive patients in CESC more suitable for immune checkpoint therapy (**Figures 2A**, **3**). We also found that the mutation frequency of the antitumor gene *PTEN* (55, 56) in the HPV-positive group of CESC was significantly lower than that in the HPV-negative group. The low mutation frequency of the two famous tumor-suppressor genes *TP53* and *PTEN* in patients in the CESC HPV-positive group indicated that these patients received more “help” in the process of fighting against tumor cells. The mutation frequencies of several tumor-suppressor genes including *FAT1*, *CDKN2A*, *FGFR3*, and *CASP8* were lower in the HPV-positive group of HNSC, and even the mutation frequencies of *FAT1*, *CDKN2A*, and *CASP8* were 0 (**Supplementary Table S4**), indicating that the biological processes regulated by these genes were not disrupted. Knocking down *FAT1* and *CASP8* separately or together resulted in enhanced cell motility and clonal development (57).

Due to significantly lower mutation frequencies of the famous tumor-suppressor gene *TP53* in the HPV-positive group of CESC and HNSC, we conjectured that the DNA damage repair mechanism was stronger in the HPV-positive group of CESC and HNSC. Therefore, we calculated the activity of 10 DNA damage-repair pathways through the ssGSEA method and examined the difference of pathway activity and other genomic instability indicators between HPV-positive and HPV-negative groups. The result was consistent with our conjecture. We did not observe changes in the genomic instability indicators in CESC, which might be that the DDR pathway was not activated as highly as that in HNSC. These results indicate that genomic instability might not be the major cause for the occurrence of CESC and HNSC in the HPV-positive group. Together with the differences in carcinogenic pathways, we found that HPV infection in CESC and HNSC allowed patients to bypass the genomic instability in the carcinogenic process and directly captured the characteristics of the active cell cycle, thereby causing abnormal proliferation (58–60). These results also remind us that the detection of *TP53* mutations cannot be fit for all the people at risk of cancer, and gene mutation testing combined with HPV status is the best way to predict the risk of HNSC because of the low *TP53* mutation frequency in HPV-positive patients. We also found that the most common mutations in *TP53* were R248Q/W (19 of 431 mutations), E285K (3 of 28 mutations), and R273C/H/S (23 of 217 mutations) for HNSC, CESC, and UCEC, respectively. The R248 in p53’s DNA-binding domain (DBD) could interact with DNA’s minor groove directly and the R248Q mutation caused conformation alterations in areas of DBD far from the mutation site (61). Tumor mutations at site E285 in the H2 region of p53 may decrease essential interactions that stabilize H2, implying that the inactivation mechanisms may be linked to the loss of local structure around H2, reducing overall stability to a meaningful degree (62). Garg et al. found that the oncogenic p53 variations R273 (R273H, R273C, and R273L) not only lose their DNA-binding capabilities but also have different structural stability, aggregation, and toxicity profiles and lead to different types of cancer pathogenesis *in vivo* (63).

It is important to explore the impact of HPV infection on the tumor microenvironment (TME), because the immune infiltration and metabolism in TME are associated with patients’ prognosis (64–66). HPV infection stimulates the immune system response which may be the reason why HPV-infected patients have better prognosis than non-infected patients in CESC, HNSC, UCEC, and STAD. The immune system of HNSC had the strongest response after being stimulated by HPV (**Figure 3**). Interestingly, CD8+ memory T cells in CESC and HNSC were both increased in HPV-positive patients, implying that HPV vaccine injection may have the potential to prevent HPV infection which leads to the occurrence of HNSC. We also found that an increase in CD8+ T cells required the cooperation of dendritic cells. Upregulated dendritic cells presented more antigens to CD8+ T cells and made them upregulated (**Figure 3**). The stromal cells were also affected by HPV in the tumor microenvironment. In the patients with HPV-positive CESC and HNSC, the reduction of multiple stromal cell types (**Figure 3**) exerted a positive effect on the prognosis of patients (31, 32). TCR and BCR increased in the HPV-positive group of HNSC without the increase of mutation load and neoantigen, implying that HPV might express viral antigens and be recognized by T cells and B cells. Notably, a general trend could be observed where significant differences in the carcinogenic process and tumor microenvironment occurred in cancers with high HPV expression levels (**Figures 2A–C**, **3**). Expression analysis revealed that HPV integration disrupted gene expression, but the upregulation of *CD274*, *PDCD1LG2*, *FOXA1*, and *TNFSF4* provided opportunities for tumor immunotherapy (**Figure 4C**). Although HPV-integrated genes were enriched in GO terms that negatively regulate immunity, the presence of HPV still irreversibly activated the cell-mediated immune response (**Supplementary Figure S2** and **Figure 3**).

Since HPV has a significant impact on the tumor microenvironment which is crucial to the chemotherapy effect of cancer patients (67–69), we analyzed whether the HPV could affect chemotherapy response. After developing HPV prediction models by transcriptome characteristics, the prediction score VIS was positively correlated with the abundance of virus expression and the correlation coefficient ranged from 0.47 to 0.96. The prediction models of the cancer types with a high correlation coefficient (R > 0.8) were extended to the GDSC data for the HPV-like propensity of the cell line. When scaled VIS reached a certain level (scaled VIS > 2), the patients were quite sensitive to the chemotherapy in TCGA (**Figure 6A**). We further studied the sensitivity of HPV-like cell lines to drugs and screened out some drugs which were associated with scaled VIS (**Figure 6C**). Although we have not collected appropriate immunotherapy data, we still analyzed the relationship between VIS and the signature of immunotherapy, and the results showed that patients with high VIS may benefit more from immunotherapy (**Figures 6D–F**). If there are suitable data in the future, we can further explore the potential application of VIS as an immunotherapy marker.

Although there was a higher occurrence of HPV infection in males with STAD or HNSC (**Supplementary Table S6**), gender was not an important factor for overall survival (**Figure 1B**). We also explored whether or not there was any difference in immune cell infiltration and drug response between males and females with HPV or without HPV for STAD and HNSC. The result showed that there was no significant difference in immune cell infiltration between males and females in HPV-positive patients, but there were some significant differences (such as for CD8+ Tcm) in HNSC HPV-negative patients (**Supplementary Table S8**). Similarly, there was no significant difference in drug response both in the HPV-positive group (Fisher’s exact test, p = 0.72) and in the HPV-negative group (Fisher’s exact test, p = 0.83). Gender cannot be a considerable factor for HPV-positive cancer.

In conclusion, we conducted a multilevel analysis of a variety of cancer types with HPV infection, including the carcinogenic process of cancer and the tumor microenvironment, and propose that the high level of HPV expression may provide references for precision medicine for related cancer patients.

## Data Availability Statement

The original contributions presented in the study are included in the article/**Supplementary Material**. Further inquiries can be directed to the corresponding authors.

## Author Contributions

Conception and design: KL, JX, SZ. Development of methodology: XY, JX, DX. Acquisition of data: XY, JX, DX, XB, HW, YL, MC, WW, ZX, DZ, LC. Analysis and interpretation of data (e.g., statistical analysis, biostatistics, computational analysis): XY, JX, DX, XB, HW, YL, MC, WW, ZX, DZ, LC. Writing, review, and/or revision of the manuscript: XY, JX, DX, XB, HW, YL, MC, WW, ZX, DZ, LC. Administrative, technical, or material support (i.e., reporting or organizing data): XY, JX, DX, KL, SZ. Study supervision: KL, JX, SZ. All authors contributed to the article and approved the submitted version.

## Funding

This work was supported by the key research and development project of Hainan Province (ZDYF2021096, ZDYF2020132, ZDYF2021SHFZ098); the National Natural Science Foundation of China (32160152, 81760553, 81960528); Hainan Provincial Natural Science Foundation of China (820RC637, 821RC695); the Open program for Key Laboratory of Tropical Translational Medicine of Ministry of Education (Hainan Medical University) (2020TTM005); Major Science and Technology Program of Hainan Province (ZDKJ202003, ZDKJ2021040); Hainan Province Clinical Medical Center and Innovation Research Fund for Graduate Students (Hyb2020-56, Hys2020-378, HYYS2021A33 ,Qhys2021-354, 202111810014, X202111810069, X202111810108).

## Conflict of Interest

The authors declare that the research was conducted in the absence of any commercial or financial relationships that could be construed as a potential conflict of interest.

## Publisher’s Note

All claims expressed in this article are solely those of the authors and do not necessarily represent those of their affiliated organizations, or those of the publisher, the editors and the reviewers. Any product that may be evaluated in this article, or claim that may be made by its manufacturer, is not guaranteed or endorsed by the publisher.

## References

[1] Hoppe-Seyler K Bossler F Braun JA Herrmann AL Hoppe-Seyler F. The HPV E6/E7 Oncogenes: Key Factors for Viral Carcinogenesis and Therapeutic Targets. Trends Microbiol (2018) 26:158–68. doi: 10.1016/j.tim.2017.07.007 28823569

[2] Mantovani F Banks L. The Human Papillomavirus E6 Protein and its Contribution to Malignant Progression. Oncogene (2001) 20:7874–87. doi: 10.1038/sj.onc.1204869 11753670

[3] Forman D de Martel C Lacey CJ Soerjomataram I Lortet-Tieulent J Bruni L. Global Burden of Human Papillomavirus and Related Diseases. Vaccine (2012) 30 Suppl 5:F12–23. doi: 10.1016/j.vaccine.2012.07.055 23199955

[4] Schiffman M Castle PE Jeronimo J Rodriguez AC Wacholder S. Human Papillomavirus and Cervical Cancer. Lancet (London England) (2007) 370:890–907. doi: 10.1016/S0140-6736(07)61416-0 17826171

[5] zur Hausen H. Papillomaviruses and Cancer: From Basic Studies to Clinical Application. Nat Rev Cancer (2002) 2:342–50. doi: 10.1038/nrc798 12044010

[6] Guimerà N Alemany L Halec G Pawlita M Wain GV Vailén JSS. Human Papillomavirus 16 is an Aetiological Factor of Scrotal Cancer. Br J Cancer (2017) 116:1218–22. doi: 10.1038/bjc.2017.74 PMC5418448 28376081

[7] Marur S D’Souza G Westra WH Forastiere AA. HPV-Associated Head and Neck Cancer: A Virus-Related Cancer Epidemic. Lancet Oncol (2010) 11:781–9. doi: 10.1016/S1470-2045(10)70017-6 PMC5242182 20451455

[8] Alemany L Cubilla A Halec G Kasamatsu E Quirós B Masferrer E. Role of Human Papillomavirus in Penile Carcinomas Worldwide. Eur Urol (2016) 69:953–61. doi: 10.1016/j.eururo.2015.12.007 26762611

[9] Singh N Hussain S Kakkar N Singh SK Sobti RC Bharadwaj M. Implication of High Risk Human Papillomavirus HR-HPV Infection in Prostate Cancer in Indian Population–a Pioneering Case-Control Analysis. Sci Rep (2015) 5:7822. doi: 10.1038/srep07822 25592643 PMC4296305

[10] Cancer Genome Atlas Research Network. Comprehensive Molecular Characterization of Urothelial Bladder Carcinoma. Nature (2014) 507:315–22. doi: 10.1038/nature12965 PMC3962515 24476821

[11] Lawson JS Glenn WK Salyakina D Delprado W Clay R Antonsson A. Human Papilloma Viruses and Breast Cancer. Front Oncol (2015) 5:277. doi: 10.3389/fonc.2015.00277 26734565 PMC4679879

[12] Gameiro SF Ghasemi F Barrett JW Koropatnick J Nichols AC Mymryk JS. Treatment-Naïve HPV+ Head and Neck Cancers Display a T-Cell-Inflamed Phenotype Distinct From Their HPV- Counterparts That has Implications for Immunotherapy. Oncoimmunology (2018) 7:e1498439. doi: 10.1080/2162402X.2018.1498439 30288365 PMC6169583

[13] Varn FS Schaafsma E Wang Y Cheng C. Genomic Characterization of Six Virus-Associated Cancers Identifies Changes in the Tumor Immune Microenvironment and Altered Genetic Programs. Cancer Res (2018) 78:6413–23. doi: 10.1158/0008-5472.CAN-18-1342 PMC6239894 30254145

[14] Cao S Wylie KM Wyczalkowski MA Karpova A Ley J Sun S. Dynamic Host Immune Response in Virus-Associated Cancers. Commun Biol (2019) 2:109. doi: 10.1038/s42003-019-0352-3 30911684 PMC6430765

[15] Baghban R Roshangar L Jahanban-Esfahlan R Seidi K Ebrahimi-Kalan A Jaymand M. Tumor Microenvironment Complexity and Therapeutic Implications at a Glance. Cell Commun Signal (2020) 18:59. doi: 10.1186/s12964-020-0530-4 32264958 PMC7140346

[16] Martincorena I Campbell PJ. Somatic Mutation in Cancer and Normal Cells. Science (2015) 349:1483–9. doi: 10.1126/science.aab4082 26404825

[17] Sanchez-Vega F Mina M Armenia J Chatila WK Luna A La KC. Oncogenic Signaling Pathways in The Cancer Genome Atlas. Cell (2018) 173:321–37.e10. doi: 10.1016/j.cell.2018.03.035 29625050 PMC6070353

[18] Iglesia MD Parker JS Hoadley KA Serody JS Perou CM Vincent BG. Genomic Analysis of Immune Cell Infiltrates Across 11 Tumor Types. J Natl Cancer Inst (2016) 108. doi: 10.1093/jnci/djw144 PMC5241901 27335052

[19] Li B Severson E Pignon J-C Zhao H Li T Novak J. Comprehensive Analyses of Tumor Immunity: Implications for Cancer Immunotherapy. Genome Biol (2016) 17:174. doi: 10.1186/s13059-016-1028-7 27549193 PMC4993001

[20] Thorsson V Gibbs DL Brown SD Wolf D Bortone DS Ou Yang T-H. The Immune Landscape of Cancer. Immunity (2019) 51:411–2. doi: 10.1016/j.immuni.2019.08.004 31433971

[21] Liu J Lichtenberg T Hoadley KA Poisson LM Lazar AJ Cherniack AD. An Integrated TCGA Pan-Cancer Clinical Data Resource to Drive High-Quality Survival Outcome Analytics. Cell (2018) 173:400–16.e11. doi: 10.1016/j.cell.2018.02.052 29625055 PMC6066282

[22] Ding Z Zu S Gu J. Evaluating the Molecule-Based Prediction of Clinical Drug Responses in Cancer. Bioinformatics (2016) 32:2891–5. doi: 10.1093/bioinformatics/btw344 27354694

[23] Bailey MH Tokheim C Porta-Pardo E Sengupta S Bertrand D Weerasinghe A. Comprehensive Characterization of Cancer Driver Genes and Mutations. Cell (2018) 173:371–85.e18. doi: 10.1016/j.cell.2018.02.060 29625053 PMC6029450

[24] Koneva LA Zhang Y Virani S Hall PB McHugh JB Chepeha DB. HPV Integration in HNSCC Correlates With Survival Outcomes, Immune Response Signatures, and Candidate Drivers. Mol Cancer Res (2018) 16:90–102. doi: 10.1158/1541-7786.MCR-17-0153 28928286 PMC5752568

[25] Knijnenburg TA Wang L Zimmermann MT Chambwe N Gao GF Cherniack AD. Genomic and Molecular Landscape of DNA Damage Repair Deficiency Across The Cancer Genome Atlas. Cell Rep (2018) 23:239–54.e6. doi: 10.1016/j.celrep.2018.03.076 29617664 PMC5961503

[26] Peng X Chen Z Farshidfar F Xu X Lorenzi PL Wang Y. Molecular Characterization and Clinical Relevance of Metabolic Expression Subtypes in Human Cancers. Cell Rep (2018) 23:255–69.e4. doi: 10.1016/j.celrep.2018.03.077 29617665 PMC5916795

[27] Aran D Hu Z Butte AJ. Xcell: Digitally Portraying the Tissue Cellular Heterogeneity Landscape. Genome Biol (2017) 18:220. doi: 10.1186/s13059-017-1349-1 29141660 PMC5688663

[28] Bonneville R Krook MA Kautto EA Miya J Wing MR Chen H-Z. Landscape of Microsatellite Instability Across 39 Cancer Types. JCO Precis Oncol (2017) 2017. doi: 10.1200/PO.17.00073 PMC5972025 29850653

[29] Love MI Huber W Anders S. Moderated Estimation of Fold Change and Dispersion for RNA-Seq Data With Deseq2. Genome Biol (2014) 15:550. doi: 10.1186/s13059-014-0550-8 25516281 PMC4302049

[30] Ang KK Harris J Wheeler R Weber R Rosenthal DI Nguyen-Tân PF. Human Papillomavirus and Survival of Patients With Oropharyngeal Cancer. N Engl J Med (2010) 363:24–35. doi: 10.1056/NEJMoa0912217 20530316 PMC2943767

[31] Valkenburg KC de Groot AE Pienta KJ. Targeting the Tumour Stroma to Improve Cancer Therapy. Nat Rev Clin Oncol (2018) 15:366–81. doi: 10.1038/s41571-018-0007-1 PMC5960434 29651130

[32] Wen Y Wang C-T Ma T-T Li Z-Y Zhou L-N Mu B. Immunotherapy Targeting Fibroblast Activation Protein Inhibits Tumor Growth and Increases Survival in a Murine Colon Cancer Model. Cancer Sci (2010) 101:2325–32. doi: 10.1111/j.1349-7006.2010.01695.x PMC11158467 20804499

[33] Whitehurst AW. Cause and Consequence of Cancer/Testis Antigen Activation in Cancer. Annu Rev Pharmacol Toxicol (2014) 54:251–72. doi: 10.1146/annurev-pharmtox-011112-140326 24160706

[34] Djureinovic D Dodig-Crnković T Hellström C Holgersson G Bergqvist M Mattsson JSM. Detection of Autoantibodies Against Cancer-Testis Antigens in non-Small Cell Lung Cancer. Lung Cancer (2018) 125:157–63. doi: 10.1016/j.lungcan.2018.09.012 30429015

[35] Gordeeva O. Cancer-Testis Antigens: Unique Cancer Stem Cell Biomarkers and Targets for Cancer Therapy. Semin Cancer Biol (2018) 53:75–89. doi: 10.1016/j.semcancer.2018.08.006 30171980

[36] Rosenberg SA. Progress in Human Tumour Immunology and Immunotherapy. Nature (2001) 411:380–4. doi: 10.1038/35077246 11357146

[37] Newton Y Novak AM Swatloski T McColl DC Chopra S Graim K. TumorMap: Exploring the Molecular Similarities of Cancer Samples in an Interactive Portal. Cancer Res (2017) 77:e111–4. doi: 10.1158/0008-5472.CAN-17-0580 PMC5751940 29092953

[38] Hudelist G Manavi M Pischinger KID Watkins-Riedel T Singer CF Kubista E. Physical State and Expression of HPV DNA in Benign and Dysplastic Cervical Tissue: Different Levels of Viral Integration are Correlated With Lesion Grade. Gynecol Oncol (2004) 92:873–80. doi: 10.1016/j.ygyno.2003.11.035 14984955

[39] Daniel B Rangarajan A Mukherjee G Vallikad E Krishna S. The Link Between Integration and Expression of Human Papillomavirus Type 16 Genomes and Cellular Changes in the Evolution of Cervical Intraepithelial Neoplastic Lesions. J Gen Virol (1997) 78( Pt 5):1095–101. doi: 10.1099/0022-1317-78-5-1095 9152428

[40] Hoadley KA Yau C Hinoue T Wolf DM Lazar AJ Drill E. Cell-Of-Origin Patterns Dominate the Molecular Classification of 10,000 Tumors From 33 Types of Cancer. Cell (2018) 173:291–304.e6. doi: 10.1016/j.cell.2018.03.022 29625048 PMC5957518

[41] Sidaway P. Immunotherapy: HLA-1 Genotype Influences Response to Checkpoint Inhibitors. Nat Rev Clin Oncol (2018) 15:66. doi: 10.1038/nrclinonc.2017.210 29297507

[42] Lei X Lei Y Li J-K Du W-X Li R-G Yang J. Immune Cells Within the Tumor Microenvironment: Biological Functions and Roles in Cancer Immunotherapy. Cancer Lett (2020) 470:126–33. doi: 10.1016/j.canlet.2019.11.009 31730903

[43] Ribas A Wolchok JD. Cancer Immunotherapy Using Checkpoint Blockade. Science (2018) 359:1350–5. doi: 10.1126/science.aar4060 PMC7391259 29567705

[44] Galloway DA Laimins LA. Human Papillomaviruses: Shared and Distinct Pathways for Pathogenesis. Curr Opin Virol (2015) 14:87–92. doi: 10.1016/j.coviro.2015.09.001 26398222 PMC4628885

[45] Zhou J Peng C Li B Wang F Zhou C Hong D. Transcriptional Gene Silencing of HPV16 E6/E7 Induces Growth Inhibition *via* Apoptosis *In Vitro* and *In Vivo* . Gynecol Oncol (2012) 124:296–302. doi: 10.1016/j.ygyno.2011.10.028 22056554

[46] Özcan-Wahlbrink M Schifflers C Riemer AB. Enhanced Radiation Sensitivity of Human Papillomavirus-Driven Head and Neck Cancer: Focus on Immunological Aspects. Front Immunol (2019) 10:2831. doi: 10.3389/fimmu.2019.02831 31849993 PMC6901628

[47] Lu C El-Deiry WS. Targeting P53 for Enhanced Radio- and Chemo-Sensitivity. Apoptosis (2009) 14:597–606. doi: 10.1007/s10495-009-0330-1 19259822

[48] Ren S Gaykalova DA Guo T Favorov AV Fertig EJ Tamayo P. HPV E2, E4, E5 Drive Alternative Carcinogenic Pathways in HPV Positive Cancers. Oncogene (2020) 39:6327–39. doi: 10.1038/s41388-020-01431-8 PMC7529583 32848210

[49] Cai W Song B Ai H. Combined Inhibition of FGFR and mTOR Pathways is Effective in Suppressing Ovarian Cancer. Am J Transl Res (2019) 11:1616–25. PMC6456542 30972187

[50] Donehower LA Soussi T Korkut A Liu Y Schultz A Cardenas M. Integrated Analysis of TP53 Gene and Pathway Alterations in The Cancer Genome Atlas. Cell Rep (2019) 28:1370–84.e5. doi: 10.1016/j.celrep.2019.07.001 31365877 PMC7546539

[51] Eischen CM. Genome Stability Requires P53. Cold Spring Harb Perspect Med (2016) 6. doi: 10.1101/cshperspect.a026096 PMC4888814 27252396

[52] Nagarajan S Rao SV Sutton J Cheeseman D Dunn S Papachristou EK. ARID1A Influences HDAC1/BRD4 Activity, Intrinsic Proliferative Capacity and Breast Cancer Treatment Response. Nat Genet (2020) 52:187–97. doi: 10.1038/s41588-019-0541-5 PMC7116647 31913353

[53] Hu G Tu W Yang L Peng G Yang L. ARID1A Deficiency and Immune Checkpoint Blockade Therapy: From Mechanisms to Clinical Application. Cancer Lett (2020) 473:148–55. doi: 10.1016/j.canlet.2020.01.001 31911080

[54] Shen J Ju Z Zhao W Wang L Peng Y Ge Z. ARID1A Deficiency Promotes Mutability and Potentiates Therapeutic Antitumor Immunity Unleashed by Immune Checkpoint Blockade. Nat Med (2018) 24:556–62. doi: 10.1038/s41591-018-0012-z PMC6076433 29736026

[55] Papa A Wan L Bonora M Salmena L Song MS Hobbs RM. Cancer-Associated PTEN Mutants Act in a Dominant-Negative Manner to Suppress PTEN Protein Function. Cell (2014) 157:595–610. doi: 10.1016/j.cell.2014.03.027 24766807 PMC4098792

[56] Chen C-Y Chen J He L Stiles BL. PTEN: Tumor Suppressor and Metabolic Regulator. Front Endocrinol (Lausanne) (2018) 9:338. doi: 10.3389/fendo.2018.00338 30038596 PMC6046409

[57] Hayes TF Benaich N Goldie SJ Sipilä K Ames-Draycott A Cai W. Integrative Genomic and Functional Analysis of Human Oral Squamous Cell Carcinoma Cell Lines Reveals Synergistic Effects of FAT1 and CASP8 Inactivation. Cancer Lett (2016) 383:106–14. doi: 10.1016/j.canlet.2016.09.014 PMC5090049 27693639

[58] Fan Y Sanyal S Bruzzone R. Breaking Bad: How Viruses Subvert the Cell Cycle. Front Cell Infect Microbiol (2018) 8:396. doi: 10.3389/fcimb.2018.00396 30510918 PMC6252338

[59] Graham SV. The Human Papillomavirus Replication Cycle, and its Links to Cancer Progression: A Comprehensive Review. Clin Sci (Lond) (2017) 131:2201–21. doi: 10.1042/CS20160786 28798073

[60] Muñoz N Bosch FX de Sanjosé S Herrero R Castellsagué X Shah KV. International Agency for Research on Cancer Multicenter Cervical Cancer Study Group. Epidemiologic Classification of Human Papillomavirus Types Associated With Cervical Cancer. N Engl J Med (2003) 348:518–27. doi: 10.1056/NEJMoa021641 12571259

[61] Ng JWK Lama D Lukman S Lane DP Verma CS Sim AYL. R248Q Mutation–Beyond P53-DNA Binding. Proteins (2015) 83:2240–50. doi: 10.1002/prot.24940 26442703

[62] Lu Q Tan Y-H Luo R. Molecular Dynamics Simulations of P53 DNA-Binding Domain. J Phys Chem B (2007) 111:11538–45. doi: 10.1021/jp0742261 PMC2522240 17824689

[63] Garg A Hazra JP Sannigrahi MK Rakshit S Sinha S. Variable Mutations at the P53-R273 Oncogenic Hotspot Position Leads to Altered Properties. Biophys J (2020) 118:720–8. doi: 10.1016/j.bpj.2019.12.015 PMC7002923 31952808

[64] Dey P Kimmelman AC DePinho RA. Metabolic Codependencies in the Tumor Microenvironment. Cancer Discov (2021) 11:1067–81. doi: 10.1158/2159-8290.CD-20-1211 PMC8102306 33504580

[65] Vitale I Manic G Coussens LM Kroemer G Galluzzi L. Macrophages and Metabolism in the Tumor Microenvironment. Cell Metab (2019) 30:36–50. doi: 10.1016/j.cmet.2019.06.001 31269428

[66] Pagès F Galon J Dieu-Nosjean M-C Tartour E Sautès-Fridman C Fridman W-H. Immune Infiltration in Human Tumors: A Prognostic Factor That Should Not be Ignored. Oncogene (2010) 29:1093–102. doi: 10.1038/onc.2009.416 19946335

[67] Nakasone ES Askautrud HA Kees T Park J-H Plaks V Ewald AJ. Imaging Tumor-Stroma Interactions During Chemotherapy Reveals Contributions of the Microenvironment to Resistance. Cancer Cell (2012) 21:488–503. doi: 10.1016/j.ccr.2012.02.017 22516258 PMC3332002

[68] Bader JE Voss K Rathmell JC. Targeting Metabolism to Improve the Tumor Microenvironment for Cancer Immunotherapy. Mol Cell (2020) 78:1019–33. doi: 10.1016/j.molcel.2020.05.034 PMC7339967 32559423

[69] Sun Y. Tumor Microenvironment and Cancer Therapy Resistance. Cancer Lett (2016) 380:205–15. doi: 10.1016/j.canlet.2015.07.044 26272180

