# Supplementary material for: Comprehensive Analysis of the Carcinogenic Process, Tumor Microenvironment, and Drug Response in HPV-Positive Cancers
Source: Front Oncol. 2022 Mar 22;12:842060. doi: 10.3389/fonc.2022.842060 (PMC8980807; doi:10.3389/fonc.2022.842060)
Supplement: Supplementary file 1 [file DataSheet_1.pdf]

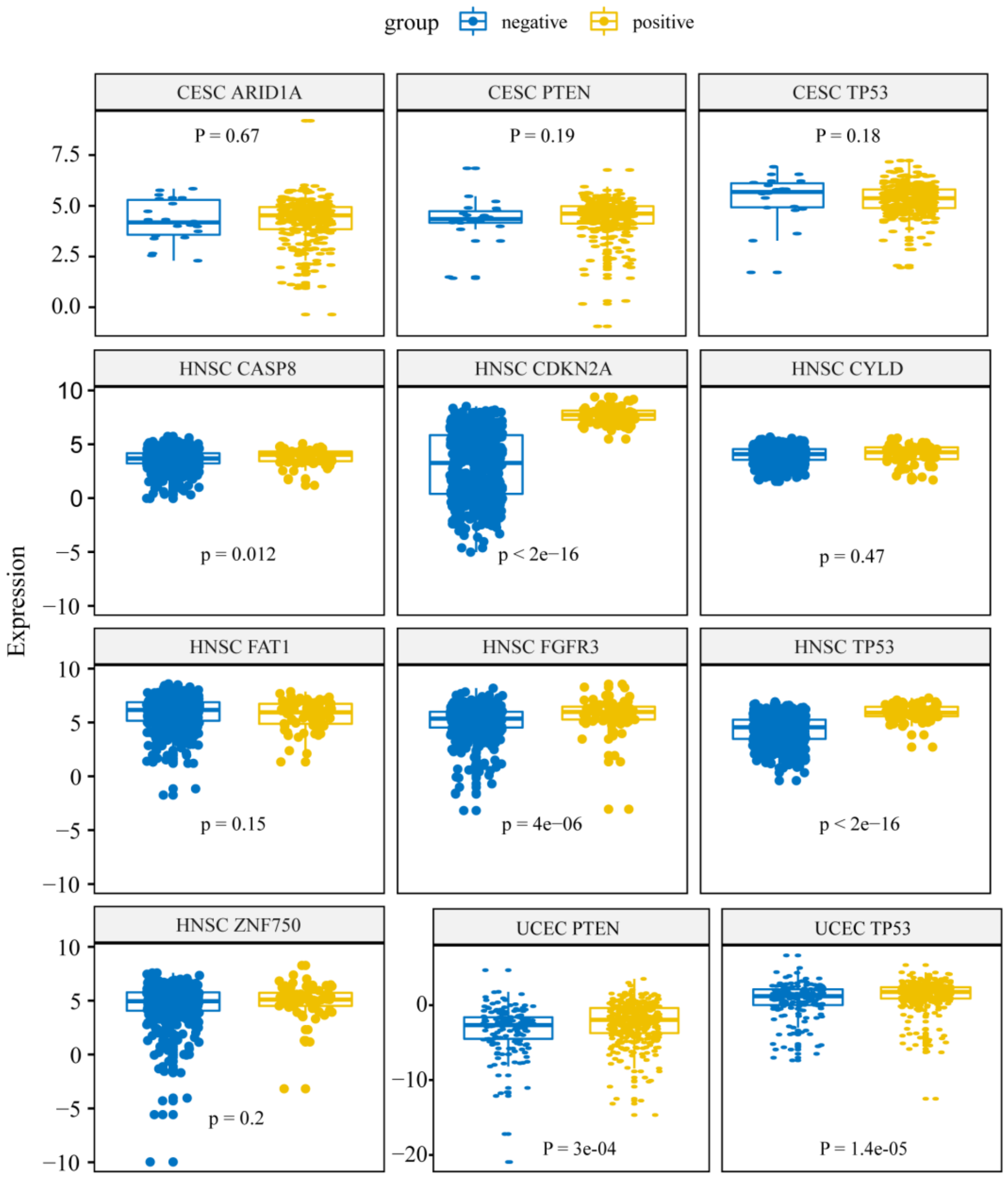

Supplementary Figure S1: Expression level of driver genes in the HPV+ and HPV- samples of CESC, HNSC and UCEC cancer types.

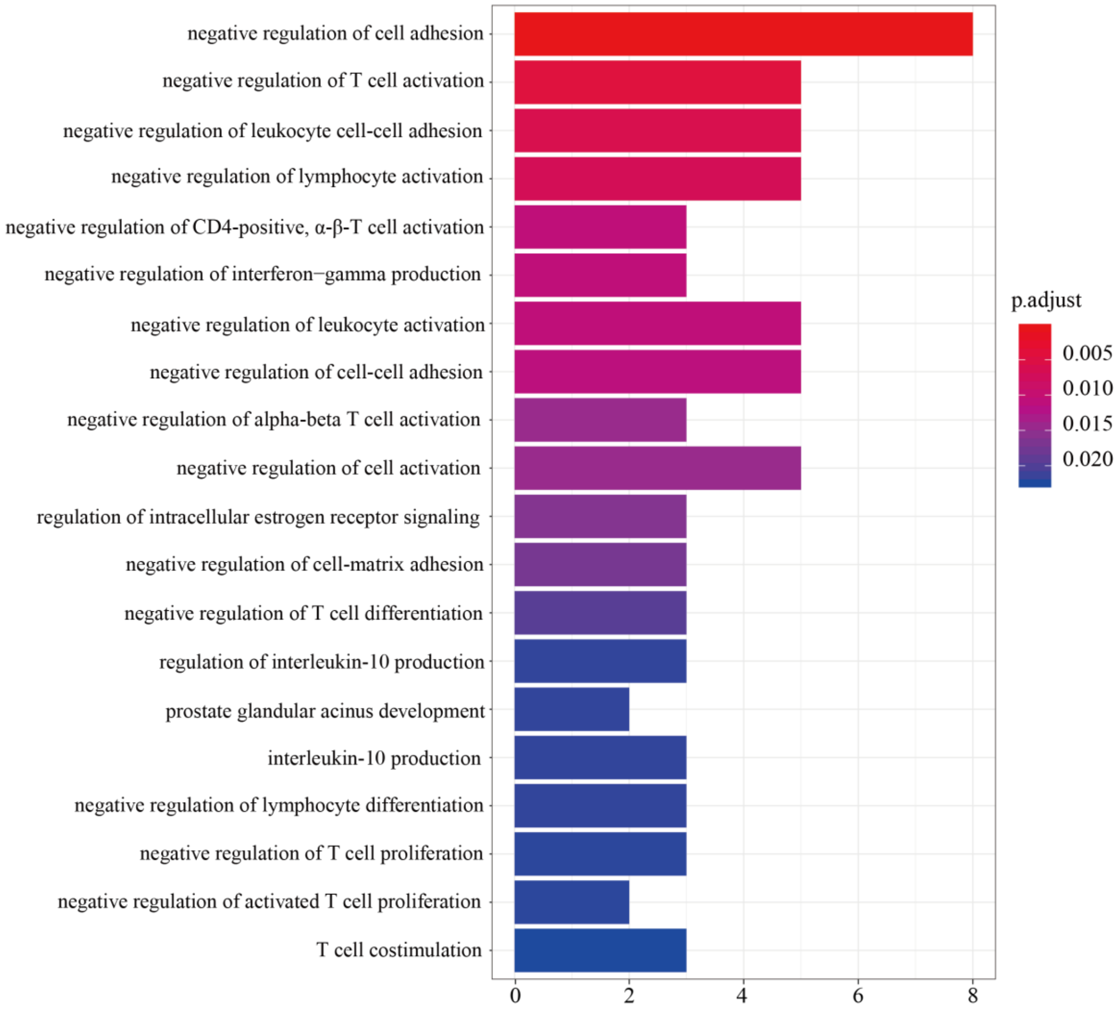

Supplementary Figure S2: Go enrichment for HPV integration genes in HNSC. Barplot showed the top 20 Gene Ontology terms for the genes integrated by HPV. The genes were most enriched for immune and cell-cell adhesion GO terms.

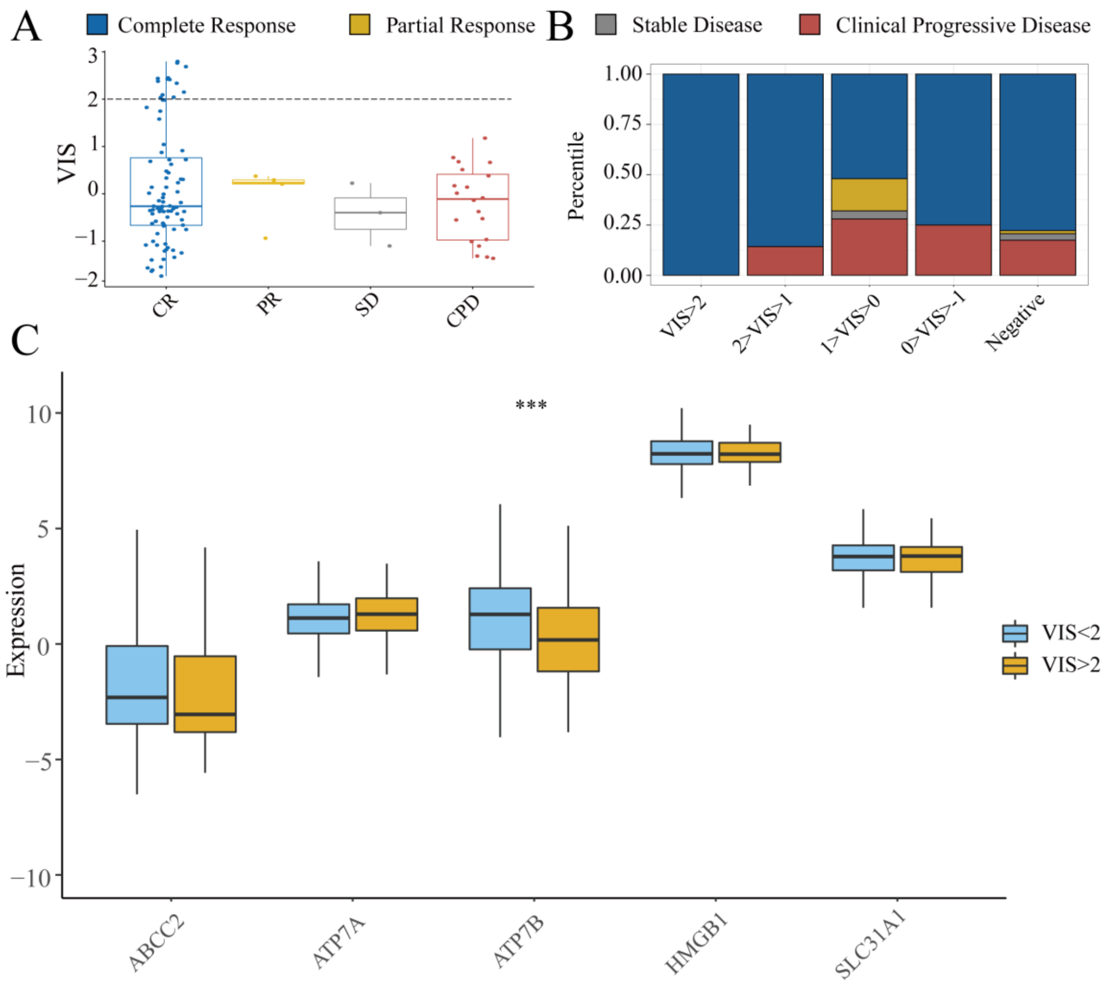

Supplementary Figure S3: the association between VIS and Platinum therapy response (A) Distribution of scaled VIS levels for TCGA stage III and stage IV samples with Platinum therapy according to the RECIST standard. (B) Proportion of Platinum therapy response in different groups segmented by VIS. (C) The differences in efflux gene expression between VIS>2 and VIS<2 groups. ***p < 0.001.
